# cPKCγ-Modulated Sequential Reactivation of mTOR Inhibited Autophagic Flux in Neurons Exposed to Oxygen Glucose Deprivation/Reperfusion

**DOI:** 10.3390/ijms19051380

**Published:** 2018-05-06

**Authors:** Rongrong Hua, Song Han, Nan Zhang, Qingqing Dai, Ting Liu, Junfa Li

**Affiliations:** Department of Neurobiology and Center of Stroke, Beijing Institute for Brain Disorders, Capital Medical University, Beijing 100069, China; rrhua@mail.ccmu.edu.cn (R.H.); songhan@ccmu.edu.cn (S.H.); nanzhang@mail.ccmu.edu.cn (N.Z.); qqingdai@mail.ccmu.edu.cn (Q.D.); tingl@mail.ccmu.edu.cn (T.L.)

**Keywords:** autophagy, conventional protein kinase Cγ, oxygen glucose deprivation, reperfusion

## Abstract

We have reported that conventional protein kinase Cγ (cPKCγ)-modulated neuron-specific autophagy improved the neurological outcome of mice following ischemic stroke through the Akt-mechanistic target of rapamycin (mTOR) pathway. However, its detailed molecular mechanism remains unclear. In this study, primary cortical neurons from postnatal one-day-old C57BL/6J *cPKCγ* wild-type (*cPKCγ*^+/+^) and knockout (*cPKCγ*^−/−^) mice suffering oxygen glucose deprivation/reperfusion (OGD/R) were used to simulate ischemia/reperfusion injury in vitro. A block of autophagic flux was observed in *cPKCγ*^+/+^ neurons under OGD/R exposure, characterized by accumulation of p62. Immunofluorescent results showed a decrease in colocalization between LC3 and Atg14 or Stx17 in cPKCγ^+/+^ neurons when compared with *cPKCγ*^−/−^ neurons after OGD/R. However, the colocalization between LC3 and Lamp2 was barely decreased, indicating the presence of autolysosomes. The larger lysotracker-positive structures were also significantly increased. These results suggest that cPKCγ-induced inhibition of autophagy occurred at the stages of autophagosome formation, Stx17 anchoring, and the degradation of autolysosomes in particular. In addition, cPKCγ-modulated phosphorylation of mTOR at Ser 2481 was dependent on the site of Ser 2448, which may have blocked autophagic flux. cPKCγ-modulated sequential reactivation of mTOR inhibited autophagic flux in neurons exposed to OGD/R, which may provide endogenous interventional strategies for stroke, especially ischemia/reperfusion injury.

## 1. Introduction

Given that recombinant tissue plasminogen activator (rtPA) thrombolysis is a recommended form of therapy for acute ischemic stroke, the mechanism of ischemia and ischemia/reperfusion injury has become a popular topic of research. Classical or conventional protein kinase Cγ (cPKCγ) is selectively expressed in neurons of the central nervous system. Studies have demonstrated that cPKCγ or its interaction partners have played a neuroprotective role in hypoxia-ischemia models in vitro and in vivo [1]. In addition, we also found that autophagy is induced in ischemic injury through the Akt-mTOR pathway, while the level of autophagy in *cPKCγ* wild-type mice is significantly lower than in knockout mice after reperfusion [2]. However, the detailed molecular mechanism underlying cPKCγ-modulated autophagy and their mediated neuroprotection against ischemic injuries remain unclear.

Autophagy (macroautophagy), which is characterized by the formation of double-membrane autophagosomes that fuse with lysosomes, is a conserved lysosome-based pathway that degrades aggregated proteins, damaged organelles, or intracellular pathogens [3]. Autophagic flux is defined as the process of formation and maturation of autolysosomes and degradation of the sequestered cargo. Autophagy is often monitored by means of the autophagy-related marker protein, microtubule-associated protein 1 light chain 3 (LC3) II in mammals [4]. LC3 II labels both phagophores and unclosed autophagosomes [5]; therefore, it may be not accurate to use LC3 II as a surrogate marker for completed autophagosomes in autophagy analysis. Moreover, a growing body of evidence suggests that LC3 also interacts with many non-autophagy-related proteins affecting other cellular pathways, and autophagy can proceed independently of it [5,6]. p62/sequestosome 1 (SQSTM1), which was the first-reported mammalian selective receptor required for aggrephagy, is specifically degraded by autophagy [7,8]. Therefore, the combined study of LC3 conversion and p62 levels can monitor the integrity of autophagic flux and measure autophagy levels.

Numerous researchers have focused on the late stage of autophagy (autolysosome formation and degradation) on the basis of studies which focused on the early stage (autophagy initiation and autophagosome formation) [9,10,11,12]. Studies have shown that, with the occurrence of autophagy, mTOR can be reactivated to induce autophagic lysosome reformation (ALR) and negative feedback inhibition of autophagy under certain conditions [9,13]. In this study, we used oxygen glucose deprivation/reperfusion (OGD/R) treatment of primary cortical neurons to simulate ischemia/reperfusion injury in vitro to understand whether cPKCγ-modulated mTOR activation can block autophagy flux and its possible mechanism.

## 2. Results

### 2.1. cPKCγ Gene Knockout Reduces the Survival of Primary Cortical Neurons after 1 h OGD/R 0–12 h Treatments through Excessive Autophagy

**Figure 1:**
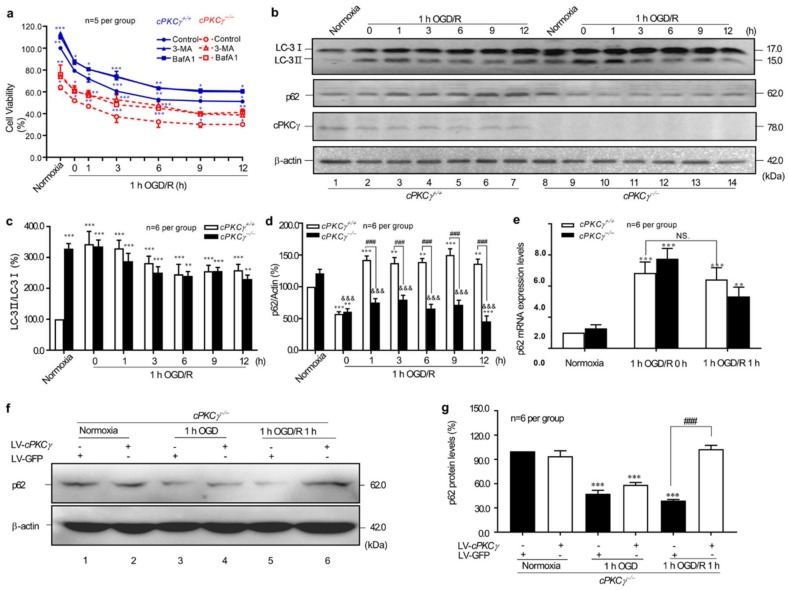
cPKCγ-mediated blocking of autophagic flux alleviates OGD/R induced neuronal injuries. (**a**) The viability of neurons under 1 h OGD/R 0–12 h exposure from *cPKCγ*^+/+^ mice was significantly higher than that from *cPKCγ*^−/−^ mice with the same treatment. Autophagy inhibitor 3-MA (5 mM) or BafA1 (100 nM) reversed the decrease; (**b**) Representative results; (**c**) The exposure of 1 h OGD/R 0–12 h could increase the LC3II/LC3I ratio in the both groups; (**d**) The protein levels of p62 were decreased in neurons under 1 h OGD exposure from both *cPKCγ*^+/+^ and *cPKCγ*^−/−^ mice. However, 1 h OGD/R 1–12 h exposure only reversed the reduced protein levels of p62 in *cPKCγ*^+/+^ neurons; (**e**) The mRNA levels of *p62* were increased in neurons after 1 h OGD/R 0–1 h injury; (**f**,**g**) showed that lentiviral transfection of *cPKCγ* into *cPKCγ*^−/−^ neurons partly reversed the decrease in p62 protein levels induced by 1 h OGD/R 1 h exposure. (* *p* < 0.05, **p < 0.01, and *** *p* < 0.001 versus corresponding control (**a**) or Normoxia group of *cPKCγ*^+/+^ neurons (**c**–**e**,**g**); ^###^ *p* < 0.001 versus corresponding group with the same treatment; ^&&&^ *p* < 0.001 versus Normoxia group of *cPKCγ*^−/−^ neurons).

As shown in Figure 1a, the viability of primary cortical neurons significantly decreased when compared with that of the baseline (normoxia, serum, and glucose-rich condition) from wild-type (WT, *cPKCγ*^+/+^) mice after 1 h OGD/R 0–12 h exposures. In addition, we found that the *cPKCγ* gene knockout (KO, *cPKCγ*^−/−^) could enhance these ischemic injuries of neurons even in the normoxic condition or 1 h OGD/R 0–12 h treatments.

To determine the role of autophagy in neuronal ischemic injury, both autophagy inhibitors 6-Amino-3-methylpurine (3-MA) and Bafilomycin A1 (BafA1) were applied. The results of Figure 1a showed that the neuronal survival rates of both groups were significantly improved under the conditions of normoxia and 1 h OGD/R 1–12 h treatments after 1 h preincubation of 3-MA (5 mM) or BafA1 (100 nM). These results suggested that both autophagy and cPKCγ knockout could enhance neuronal ischemic injuries under OGD/R conditions.

### 2.2. cPKCγ Partly Blocks Autophagic Flux in Neurons after 1 h OGD/R 0–12 h Treatments

To further validate the effect of cPKCγ on autophagy, the changes of LC3 conversion and p62 expression levels were observed in 1 h OGD/R 0–12 h-treated neurons. In Figure 1b,c, the ratio of LC3 II/LC3 I in *cPKCγ*^−/−^ neurons was higher than that of *cPKCγ*^+/+^ neurons under normoxic condition; in the condition of 1 h OGD/R 0–12 h, the ratio of LC3 II/LC3 I in both groups maintained a high level, and no significant difference in LC3 I cleavage was observed between *cPKCγ*^−/−^ and *cPKCγ*^+/+^ neurons. Unlike the changes of LC3 I cleavage, the p62 expression levels significantly decreased in both groups following 1 h OGD/R 0 h exposure (Figure 1c,d). The p62 expression levels significantly increased in *cPKCγ*^+/+^ neurons, but the low levels of p62 were maintained in *cPKCγ*^−/−^ neurons after 1 h OGD/R 1–12 h treatments. These higher conversion ratios of LC3 I to LC3 II, in combination with the decrease in p62 expression levels, suggested the existence of higher levels of autophagic flux in *cPKCγ*^−/−^ neurons after 1 h OGD/R 0–12 h treatments.

Interestingly, p62 protein accumulated in *cPKCγ*^+/+^ neurons after 1 h OGD/R 1–12 h, but this was not a consequence of higher *p62* mRNA expression (Figure 1e). These results supported the hypothesis that it was not an increase in transcription but rather a reduction in degradation through autophagic pathways. In addition, we also found that Lentivirus-based *cPKCγ* transfection into *cPKCγ*^−/−^ neurons could reverse the decrease in p62 protein levels after 1 h OGD/R 1 h treatments (Figure 1f,g). Collectively, these results suggest that cPKCγ may inhibit autophagic flux in neurons exposed to 1 h OGD/R 0–12 h.

### 2.3. Effects of cPKCγ on Autolysosome Formation and Degradation in Neurons after 1 h OGD/R 0–1 h Treatments

**Figure 2:**
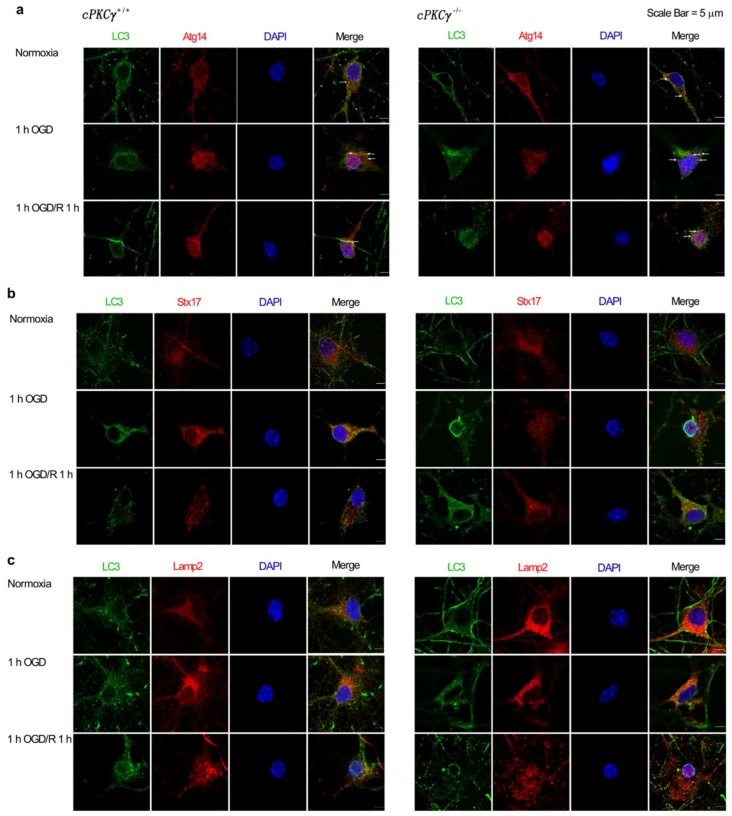

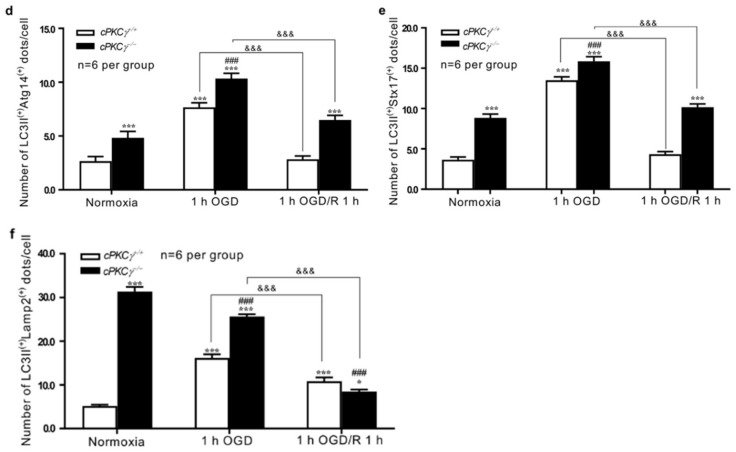
Effects of cPKCγ on autophagosome and autolysosome formation. (**a**,**d**) and (**b**,**e**) showed the colocalizations between LC3 and Atg14 or Stx17 were increased in *cPKCγ*^+/+^ and *cPKCγ*^−/−^ neurons after 1 h OGD/R 0 h exposure, indicating higher levels of autophagosome formation and Stx17 anchoring. However, the colocalizations were reduced in *cPKCγ*^+/+^ neurons (not in *cPKCγ*^−/−^ neurons) after 1 h OGD/R 1 h exposure; (**c**,**f**) The colocalization of LC3 and Lamp2 was increased in *cPKCγ*^+/+^ neuron exposed to 1 h OGD/R 0–1 h. (* *p* < 0.05 and *** *p* < 0.001 versus Normoxia group of *cPKCγ*^+/+^ neurons; ^###^ *p* < 0.001 versus Normoxia group of *cPKCγ*^−/−^ neurons; ^&&&^ *p* < 0.001 versus corresponding group; Arrows, representative colocalization; Scale bar, 5 μm).

Autophagy related 14 (Atg14) is an autophagosomal precursor marker. Syntaxin 17(Stx17), contained in soluble *N*-ethylmaleimide-sensitive factor-attachment protein receptors (SNARE) complex, was required for autophagosome tether and fusion with lysosomes. As a lysosome marker, lysosome-associated membrane protein 2 (Lamp2) will co-localize with LC3 after the formation of autolysosomes. To determine the effect of cPKCγ on autophagosome and autolysosome formations and degradations in 1 h OGD/R 0–1 h-treated neurons, the co-immunofluorescent staining of LC3 with Atg14, Stx17, or Lamp2 was observed. The colocalizations between LC3 and Atg14, Stx17, or Lamp2 were increased in *cPKCγ*^+/+^ and *cPKCγ*^−/−^ neurons after 1 h OGD/R 0 h exposure, indicating higher autophagy levels (Figure 2). However, the colocalizations between LC3 and Atg14 or Stx17 were only reduced in *cPKCγ*^+/+^ neurons after 1 h OGD/R 1 h exposure, suggesting the over-activated autophagic flux had been alleviated (Figure 2a,b,d,e). Interestingly, the colocalizations between LC3 and Lamp2 were barely decreased in *cPKCγ*^+/+^ neurons exposed to 1 h OGD/R 1 h, indicating the presence of numerous autolysosomes (Figure 2c,f). These results suggested that cPKCγ may inhibit autophagosome formation, Stx17 anchoring, and aberrant autolysosome degradation in neurons after 1 h OGD/R 1 h exposure.

**Figure 3:**
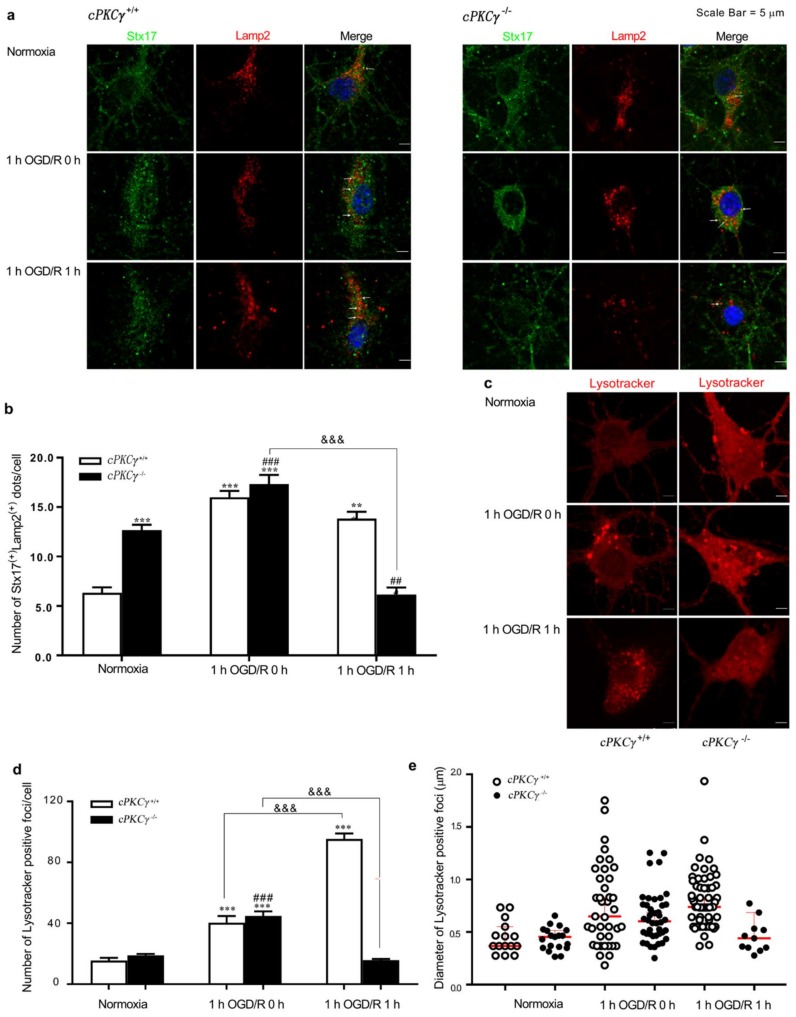
Effects of cPKCγ on autolysosome degradation. (**a**,**b**) showed the colocalization of Stx17 with Lamp2 was increased in *cPKCγ*^+/+^ and *cPKCγ*^−/−^ neurons after 1 h OGD/R 0 h exposure, indicating higher levels of autolysosome formation. The colocalization of Stx17 and Lamp2 was also increased in *cPKCγ*^+/+^ neuron exposed to 1 h OGD/R 1 h, suggesting an autophagic flux blockage in the autolysosome phase; (**c**–**e**) showed the number and diameter of Lysotracker-positive structures were significantly increased in neurons of *cPKCγ*^+/+^ neuron exposed to 1 h OGD/R 0–1 h. (** *p* < 0.01 and *** *p* < 0.001 versus Normoxia group of *cPKCγ*^+/+^ neurons; ^##^ *p* < 0.01 and ^###^ *p* < 0.001 versus Normoxia group of *cPKCγ*^−/−^ neurons; ^&&&^ *p* < 0.001 versus corresponding group; *n* = 6 per group; Arrows, representative co-localization; Scale bar, 5 μm).

There were also more Lamp2-positive foci that colocalized with Stx17 in *cPKCγ*^+/+^ neurons after 1 h OGD/R 1 h exposure compared with normoxic conditions (Figure 3a,b), suggesting the accumulation of autolysosomes. In addition, we found that the number and diameter of lysotracker-positive foci per cell, which were probably acidic autolysosomes, significantly increased in *cPKCγ*^+/+^ neurons after 1 h OGD/R 0–1 h treatments when compared with that of normoxic conditions (Figure 3c–e). These results suggest that cPKCγ may be involved in the blocking of autophagic flux, predominantly in autolysosome degradation.

### 2.4. Effects of cPKCγ on mTOR Reactivation in 1 h OGD/R 1 h-Treated Neurons

**Figure 4:**
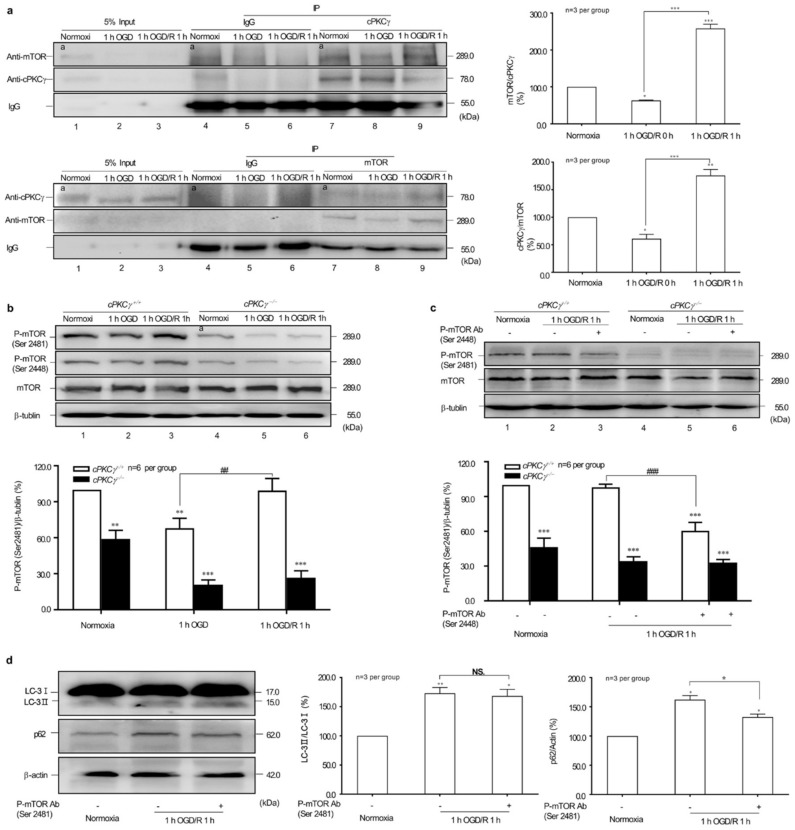
cPKCγ reactivates mTOR and alleviates autophagy in neurons under 1 h OGD/R 1 h exposure. (**a**) The representative result revealed that cPKCγ and mTOR could be coimmunoprecipitated from the samples of non-treated cortical neurons or 1 h OGD/R 0–1 h-treated neurons; (**b**) The phosphorylation levels of mTOR at Ser 2481 and 2448 in the neurons from *cPKCγ*^+/+^ mice were decreased in cPKCγ^+/+^ neurons under 1 h OGD exposure and increased after reperfusion. The phosphorylation levels of mTOR at site of Ser 2481 and 2448 were significantly lower in cPKCγ^−/−^ neurons at conditions of normoxia and 1 h OGD/R 0–1 h treatments; (**c**) Neutralizing antibody against p-mTOR at Ser 2448 offset the increase of phosphorylation levels of mTOR at Ser 2481 induced by 1 h OGD/R 1 h exposure; (**d**) Neutralizing antibody against p-mTOR at Ser 2481 unblocked autophagic flux and reduced the p62 accumulation. (* *p* < 0.05, ** *p* < 0.01 and *** *p* < 0.001 versus Normoxia group; ^##^ *p* < 0.01 and ^###^ *p* < 0.001 versus corresponding group; ^NS^ *p* > 0.05 versus corresponding group).

To elucidate the possible molecular mechanism of cPKCγ on blocking autophagic flux, the coimmunoprecipitation of cPKCγ with mTOR was first conducted in 1 h OGD/R 0–1 h-treated neurons. cPKCγ and mTOR could reciprocally coimmunoprecipitate in neurons under normoxic and especially 1 h OGD/R 1 h conditions (Figure 4a). These results suggested that there was a specific interaction between cPKCγ and mTOR in neurons.

To determine the essential biological role of cPKCγ in mTOR phosphorylation and reactivation, the phosphorylated status of mTOR at Serine (Ser) 2481 and 2448 sites were observed both in *cPKCγ*^+/+^ and *cPKCγ*^−/−^ neurons. The results showed that the phosphorylation levels of mTOR at Ser 2481 were decreased significantly in *cPKCγ*^−/−^ neurons when compared with that of *cPKCγ*^+/+^ neurons at conditions of normoxia and 1 h OGD/R 0–1 h treatments (Figure 4b). Similar changes in cPKCγ-dependent phosphorylation of mTOR at Ser 2448 were also observed in neurons, suggesting that the reactivation of mTOR was partly dependent on cPKCγ. In addition, we found that the levels of P-mTOR at Ser 2481 decreased significantly in 1 h OGD/R 1 h-treated *cPKCγ*^+/+^ (not *cPKCγ*^−/−^) neurons by adding antibodies against Ser 2448 site of P-mTOR (Figure 4c). These results suggested that cPKCγ may have modulated the phosphorylation of mTOR Ser 2481 through its effect on the Ser 2448 site in 1 h OGD/R 1 h-treated neurons.

To explore the effect of phosphorylated mTOR at Ser 2481 on autophagy, we monitored the LC3II/LC3I ratio and the p62 protein levels in neurons after Ser 2481 P-mTOR antibody neutralization treatment. The p62 accumulation in 1 h OGD/R 1 h-treated neurons was reversed by adding antibodies against Ser 2481 site of P-mTOR (Figure 4c). Combined with the similar LC3II/LC3I ratio, we can conclude that the phosphorylation of mTOR Ser 2481 may contribute to the impairment of autophagic flux.

## 3. Discussion

Autophagy is a double-edged sword in the process of death of nervous system cells [14,15,16]. Basal autophagy is particularly important for the removal of excess proteins in neurons that have no dividing properties, but the abnormal basal autophagy was linked to neurodegenerative diseases [17]. We have reported that a low level of autophagy was maintained in neurons of *cPKCγ*^+/+^ mice at baseline and a higher level of autophagy in neurons of *cPKCγ*^−/−^ mice [2]. In this study, we further found that the LC3 conversion in *cPKCγ*^−/−^ neurons was higher than that in *cPKCγ*^+/+^ neurons, and the phosphorylation level of mTOR at Ser 2481, as the auto-phosphorylation site described above, was lower than that of wild-type under the conditions of normoxia and 1 h OGD/R 0–1 h treatments. The p62 accumulation was associated with autophagy inhibition (reduction of autophagosome formation and restriction of autolysosome degradation) in *cPKCγ*^+/+^ neurons after 1 h OGD/R 1 h treatments. The inhibition of autophagic flux was attenuated with lower phosphorylation levels of mTOR at Ser 2481 in *cPKCγ*^−/−^ neurons after 1 h OGD/R 1 h. In addition, we found that cPKCγ could reciprocally coimmunoprecipitate with mTOR, especially under conditions of 1 h OGD/R 1 h. The phosphorylation levels of mTOR at Ser 2481 were affected by the neutralization of antibody against P-Ser 2448 mTOR. These results suggest that cPKCγ-modulated mTOR phosphorylation and reactivation might be involved in blocking autophagic flux during 1 h OGD/R 1 h treatments.

mTOR forms the catalytic subunit of two structurally and functionally distinct complexes classified as the mTOR complex (mTORC) 1 and mTORC2 [18]. Studies have shown that initiation of autophagy is controlled by the ULK1-Atg13-FIP200 complex, which is inhibited by mTOR kinase under nutrient replete conditions [19]. mTOR phosphorylation and reactivation can inhibit autophagy. In this study, the colocalization of LC3 with Atg14 or Stx17 was reduced in *cPKCγ*^+/+^ neurons after 1 h OGD/R 1 h treatments, suggesting the involvement of cPKCγ in regulating autophagosome formation and its subsequent steps.

In recent years, several researchers have focused on the following aspect about mTOR in autophagy: mTOR may be re-phosphorylated and inhibit autophagy after prolonged nutrient starvation or reperfusion but is dependent on amino acids [13,20]. The reoxygenation and nutrient-rich conditions in our study may give sufficient exogenous amino acids to cells for activating mTOR. The reactivated mTOR attenuated autophagy and directly triggered ALR [9]. Interestingly, proto-lysosome budding from autolysosomes in ALR only contained autolysosomal membrane components and lacked components from autophagosomes [9]. In this study, p62 protein accumulated, and the number of acidified vesicle structures whose diameters were significantly larger than baseline recordings were increased in neurons after 1 h OGD/R 1 h injury. Therefore, we speculate that, as the ALR process occurs in OGD/R, p62 protein may remain in the autolysosomes pre- or post-budding rather than being quickly degraded by hydrolysis. Further investigation is required to better understand the fate of the proteins that escape from enzymatic hydrolysis of the autolysosome.

**Figure 5:**
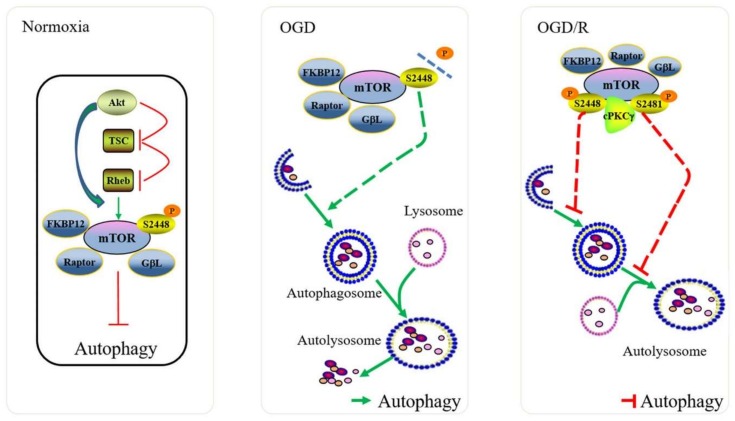
Reactivation of mTOR blocks autophagic flux and inhibits the degradation of autolysosome in neurons under 1 h OGD/R 1 h exposure. Initiation of autophagy was controlled by the ULK1, which was inhibited by mTOR kinase under nutrient replete conditions [2]. In this study, a low level of autophagy was maintained in neurons of *cPKCγ*^+/+^ mice under normoxic condition. The levels of autophagy were increased in harmony with dephosphorylation of mTOR in *cPKCγ*^+/+^ neurons after 1 h OGD treatment, whereas reperfusion was associated with Ser 2481 P-mTOR phosphorylation and reactivation. The reactived mTOR blocked autophagic flux mostly in the autolysosome degradation in neurons exposed to 1 h OGD/R 1 h.

In summary, cPKCγ participates in the inhibition of autophagic flux in neurons after 1 h OGD/R 1 h treatment, which is characterized by p62 accumulation, reduction of autophagosome formation, or restriction of autolysosome degradation. One possible mechanism involves cPKCγ-mediated phosphorylation of mTOR at Ser 2481, dependent on Ser 2448, that triggers mTOR reactivation following inhibition of autophagy (Figure 5). The specific mechanisms of cPKCγ-dependent mTOR reactivation and its effects on blocking autophagic flux predominantly in the mature autolysosome phase merits additional study. Understanding the regulation of cPKCγ on autophagic flux may reveal a new horizon in PKC study and may eventually provide endogenous interventional strategies for stroke, especially ischemia/reperfusion injury.

## 4. Materials and Methods

All animal procedures were performed in strict accordance with the recommendations in the Guide for the Care and Use of Laboratory Animals of the National Institutes of Health. All experimental protocols were approved by experimental animal ethics committee of Capital Medical University (AEEI-2014-114; 4 December 2014).

### 4.1. Cell Culture and Transfection

The primary cortical neurons were prepared from wild-type and transgenic (genotype *cPKCγ*^−/−^) C57BL/6J mice. The postnatal <24 h mice were disinfected with methanol and decapitated promptly. The cortex was separated, cut into small pieces of 0.2 cm^3^, and cultured in Dulbecco’s Modified Eagle Medium (G11995500BT, Gibco, Beijing, China), which contained 10% horse serum (16050-122, Gibco, Grand Island, NY, USA), 10% fetal bovine serum (10099-141, Gibco, Grand Island, USA), 1% penicillin-streptomycin solution (15070063, Life Technologies, Carlsbad, ON, Canada), and 0.25% l-glutamine (25030-081, Gibco, Grand Island, USA) for 4 h. Subsequently, the neurons were cultured in Neurobasal Medium (21103-040, Gibco, Grand Island, USA) containing 2% B27 supplement (17504-044, Gibco, Grand Island, USA) that was changed completely every 72 h. Cell transfection was performed using lentiviral vectors containing *cPKCγ* gene (Genepharma, Suzhou, China), according to protocols provided by the manufacturer.

### 4.2. Oxygen Glucose Deprivation/Reperfusion (OGD/R) of Primary Cortical Neurons

The OGD/R model was used to simulate ischemia/reperfusion injury in vitro. As described above, a portion of the neurons cultured for eight days were subjected to OGD/R [21]. The terms of OGD treatment were glucose-free DMEM (11966-025, Gibco, Grand Island, USA) and 5% CO_2_/2% O_2_/93% N_2_ for 1 h. The terms of reperfusion treatment were Neurobasal Medium, which contained 2% B27 supplement and 5% CO_2_/21% O_2_/74% N_2_ for 1, 3, 6, 9, or 12 h, respectively.

### 4.3. Antibodies

Anti-Atg14 (5504S, Cell Signaling, Danvers, MA, USA), Anti-LAMP2 (PRS3627, Sigma-Aldrich, St. Louis, MO, USA), Anti-LAMP2 (ab13524, Abcam, Cambridge, UK), Anti-LC3 (SAB1305552, Sigma-Aldrich), Anti-LC3 (66139-1-lg, Proteintech, Rosemont, IL, USA), Anti-mTOR (#2972, Cell Signaling), Anti-Phospho-mTOR (Ser2481) (#2974S, Cell Signaling), Anti-Phospho-mTOR (Ser2448) (#5536, Cell Signaling), Anti-p62 (ab155686, Abcam), Anti-STX17 (HPA001204, Sigma-Aldrich).

### 4.4. Cell Viability Test

The survival rate of primary cortical neurons was detected by CellTiter 96 Aqueous One Solution Cell Proliferation Assay (G3580, Promega, Madison, WI, USA) according to the manufacturer’s instructions.

### 4.5. Immunoprecipitation Assays

Whole cell lysates used for immunoprecipitation were prepared in IP buffer A (50 mM Tris-HCl (pH 7.5), 2 mM EDTA, 2 mM EGTA, 1 mM DTT, 50 mM KF, 5 M iodoacetamide, and a protease inhibitor mixture( and then incubated with specific or IgG antibody at 4 °C overnight. Washed Protein G beads and the lysates were transferred into a spin column from Protein G Immunoprecipitation Kit (1002008482, Sigma-Aldrich, St. Louis, MO, USA) and incubated at 4 °C overnight. The buffer (48 L Glycine, 2 L Tris-HCl (pH 8.0), 10 L loading buffer) was added to the beads and centrifuged. The flow-through was collected and heated at 95 °C for 5 min and processed for immunoblotting.

### 4.6. Immunofluorescence

Cells were fixed using 4% paraformaldehyde for 30 min at 25 °C, washed four times with PBS, and then blocked with buffer (8% goat serum + 0.2% Triton X100 (101875278, Sigma-Aldrich, St. Louis, MO, USA) in PBS) for 90 min at 37 °C. Cells were stained using desired antibodies at 4 °C overnight, washed with PBS, incubated with secondary antibodies for 1 h, and then mounted using ProLong Gold antifade Mountant with DAPI (P36934, Life Technologies, Carlsbad, ON, Canada). Lysotracker red DND-99 (L7528, Invitrogen, Eugene, OR, USA; 50 nM added for 30 min before fixation) were used to stain the acid structure, especially lysosomes for detection by confocal microscopy at 577/590 nm (Excitation/Emission). Cells were imaged using a Leica SP8 microscope with 63 × 1.4 Numerical Aperture (NA) oil objective lens (Leica, Wetzlar, Germany).

### 4.7. RNA Extraction and Real-Time Quantitative PCR

The neurons were disrupted in QIAzol Lysis Reagent (1023537, Qiagen, Hiden, Germany), and total RNA was extracted and quantified using the RNeasy Lipid Tissue Mini Kit (1023539, Qiagen, Hiden, Germany). RNA was reverse transcribed into cDNA by using Transcriptor First Strand cDNA Synthesis Kit (04896866001, Roche Life Sciences, Indianapolis, IN, USA). The following primers were used: *p62* forward 5′-TGTGGAACATGGAGGGAAGAG-3′, *p62* reverse 5′-TGTGCCTGTGCTGGAACTTTC-3′, *ywhaz* (as control) forward 5′-GAAGACGGAAGGTGCTGAG-3′, *ywhaz* reverse 5′-GACTTTGCTTTCTGGTTGC-3′. Real-time quantitative PCR was performed in a 20 μL reaction volume (1 μg cDNA, 1 μL primer, 10 μL Power SYBER Green PCR Master Mix (Life Technologies, Warrington, UK)), and 8 μL RNA-free water on the Applied Biosystems 7500 fast real-time PCR system (Life Technologies, Foster City, CA, USA). Triplicate reactions were performed for each sample and data were analyzed using delta-delta method (ratio, 2^−(Δ*C*T sample − Δ*C*T control)^) to compare relative mRNA levels. Real-time quantitative PCR cycling conditions were as follows: denaturation at 50 °C for 2 min and 95 °C for 10 min, followed by 40 cycles at 95 °C for 15 s, annealing/extension at 60 °C for 1 min.

### 4.8. Immunoblotting

Whole cell lysates used for immunoblotting were prepared in buffer (50 mM Tris-HCl (pH 7.5), 2 mM EDTA, 2 mM EGTA, 1 mM DTT, 50 mM KF, 2% SDS, and a protease inhibitor mixture) on ice and subjected to sodium dodecyl sulfate polyacrylamide gel electrophoresis (SDS-PAGE). The blots were blocked in 5% milk (Skim milk powder, LP0031, Oxiod, Hampshire, UK), incubated with primary antibody for 1–24 h. Membranes were washed three times for 10 min with TBS-0.05% Tween (pH 7.5), incubated with secondary antibodies for 1 h, washed again, and imaged by using Fusion-Capt Advance software on FUSION FX (Vilber Lourmat, Collégien, France).

### 4.9. Statistical Analysis

Data were expressed as mean ± SEM. Statistical analysis was performed using two-way analysis of variance (ANOVA), followed by the Bonferroni multiple comparison test with the software GraphPad Prism version 7.00 (GraphPad Software, La Jolla, CA, USA). A value of *p* < 0.05 was considered statistically significant.

## Abbreviations

cPKCγ	Conventional Protein Kinase Cγ
rtPA	recombinant tissue Plasminogen Activator
LC3	Microtubule-associated Protein 1 Light Chain 3
MCAO/R	Middle Cerebral Artery Occlusion/Reperfusion
mTOR	Mechanistic Target of Rapamycin
OGD/R	Oxygen Glucose Deprivation/Reperfusion
SQSTM1	Sequestosome 1
ALR	Autophagic Lysosome Reformation
BafA1	Bafilomycin A1
3-MA	6-Amino-3-methylpurine
